# Advanced Hepatocellular Carcinoma With Hepatic Arterioportal Shunts: Combination Treatment of Transarterial Chemoembolization With Apatinib

**DOI:** 10.3389/fmolb.2020.607520

**Published:** 2020-12-04

**Authors:** Tao Sun, Yanqiao Ren, Xuefeng Kan, Lei Chen, Weihua Zhang, Fan Yang, Chuansheng Zheng

**Affiliations:** ^1^Department of Radiology, Union Hospital, Tongji Medical College, Huazhong University of Science and Technology, Wuhan, China; ^2^Hubei Province Key Laboratory of Molecular Imaging, Wuhan, China

**Keywords:** transarterial chemoembolization, apatinib, advanced hepatocellular carcinoma, hepatic arterioportal shunts, efficacy, safety

## Abstract

**Object:** This study aimed to compare the efficacy and safety of transarterial chemoembolization (TACE) combining with apatinib (TACE-apatinib) and TACE-alone for patients with advanced hepatocellular carcinoma (HCC) with hepatic arterioportal shunts (APS).

**Materials and Methods:** This retrospective study evaluated the medical records of patients with advanced HCC with APS who underwent TACE-apatinib or TACE-alone from June 2015 to January 2019. The occlusion of the shunt was performed during the TACE procedure. The time to tumor progression (TTP) and overall survival (OS) of study patients were evaluated. The modified Response Evaluation Criteria in solid tumors (mRECIST) was used to evaluate the treatment response. The apatinib-related adverse events were recorded.

**Results:** Fifty-eight patients were included in this study. Twenty-seven patients underwent the treatment of TACE-apatinib, and 31 received TACE-alone treatment. The median overall survival (OS) and median time of tumor progression (TTP) in the TACE-apatinib group were significantly longer than those of the TACE-alone group (OS: 12.0 vs. 9.0 months, *P* = 0.000; TTP: 9.0 vs. 5.0 months, *P* = 0.041). Multivariate analysis revealed that TACE-apatinib was a protective factor for OS, and there was no independent risk factor for TTP. In the TACE-apatinib group, the grade 3 apatinib-related adverse events occurred in four patients.

**Conclusion:** TACE-apatinib was an efficacious and safe treatment for patients with advanced HCC with APS, and apatinib improved the efficacy of TACE in the treatment of these patients.

## Introduction

Hepatocellular carcinoma (HCC) is the fifth most common cancer in the world and is one of the most prevalent causes of tumor-related death (Bray et al., 2018). About 35–40% of all HCC patients are diagnosed when the disease has reached an advanced stage (Barcelona Clinic Liver Cancer [BCLC] stage C) owing to an absence of routine screening protocols and to the fact that the disease is often asymptomatic in its early stages, limiting patient treatment options (Forner et al., 2010). These patients must thus rely on palliative therapies, such as sorafenib treatment to prolong their survival (Forner et al., 2010). Recently, many studies have shown that apatinib, which is a newly developed inhibitor of vascular endothelial growth factor receptor-2 (VEGFR-2) (Ding et al., 2013), exhibits encouraging antitumor activities and tolerable toxicities when used to treat advanced HCC (Wang and Tang, 2018; Xue et al., 2018; Yang and Qin, 2018).

Hepatic arterioportal shunts (APS) is common in patients with HCC (Wu et al., 2018), affecting up to 60% of these patients (Okuda et al., 1977). The presence of APS has an adverse effect on patient prognosis and increases the incidence of complications, such as esophagus varicose rupture, refractory ascites, and hepatic encephalopathy (Velazquez et al., 2003; Lencioni et al., 2016). APS can not only damage the liver function and aggravate portal hypertension in HCC patients, but can also easily lead to the spread and metastasis of HCC (Murata et al., 2009). Additionally, APS can affect the safety of transcatheter arterial chemoembolization (TACE), as lipiodol can flow through the fistula and thereby access normal hepatic and pulmonary tissues (Ziessman et al., 1984). The standard treatment of APS relies upon the blocking of these shunts using an appropriate embolic material, which can be carried out via the TACE procedure (Kim et al., 2007).

TACE is a standard treatment for BCLC stage B HCC patients based on current BCLC guidelines European Association for the Study of the Liver, 2018). However, some previous studies reported that TACE is beneficial for patients with advanced HCC (Bai et al., 2013; Choi et al., 2013; Zhao et al., 2013). Recently, an increasing number of studies have demonstrated that a combination of TACE with apatinib treatment (TACE-apatinib) may prolong the survival of selected patients with BCLC stage-C HCC (Lu et al., 2017; Yang et al., 2019; Zhao et al., 2020). To our best knowledge, there have been few reports regarding the use of TACE-apatinib as a treatment modality in advanced HCC patients with APS. As such, we conducted a retrospective study to evaluate the efficacy and safety of TACE-apatinib for the treatment of advanced HCC with APS.

## Materials and Methods

### Patient Selections

Approval for this retrospective study was obtained from the institutional review board of our hospital. Between June 2015 and January 2019, 27 advanced HCC patients with APS who underwent TACE-apatinib were included in this study. Prior to initial TACE procedure, patients had been diagnosed with advanced HCC with APS via abdominal contrast-enhanced computed tomography (CT) or magnetic resonance (MRI). Digital subtraction angiography (DSA) was used to confirm APS during the TACE procedure. A written informed consent was obtained from all patients prior to treatment. The inclusion criteria for this study were as follows: (1) patients were diagnosed with HCC based on the guidelines of the European Association for the Study of Liver or the American Association for the Study of Liver Disease; (2) patients were staged at BCLC-C in accordance with the BCLC system; (3) patients were diagnosed with APS via medical imaging; (4) patients with liver function graded at Child-Pugh A or B; (5) patients had the Eastern Cooperative Group Performance Status (ECOG) score of patients were 0–2. The exclusion criteria of this study were as follows: (1) patients who had main portal vein obstruction; (2) patients with a poor performance status (ECOG > 2); (3) patients with significant extra-hepatic disease; (4) patients who had serious medical comorbidities, such as severe dysfunction of the liver, kidney, lung, or heart; (5) patients with massive ascites.

### TACE Procedure

The TACE procedure was performed by operators with a minimum of 5 years of experience. First, angiography of the hepatic common artery was used to identify the location, severity and direction of the vessels of APS. Then, a 5-F catheter (Cook, Bloomington, Indian, USA) or a 3-F microcatheter (Progreat, Terumo, Tokyo, Japan) was advanced into the feeding artery of APS. Polyvinyl alcohol particles (500–1,000 um, Cook, USA) that were mixed with contrast media (Hengrui Pharmaceutical Co. Ltd, Jiangsu, China) were then injected to block the APS. An arteriography was then performed to confirm the occlusion of APS. Depending on tumor size and liver function, 2–20 mL of lipiodol (Lipiodol Ultrafluido, Guerbet, France) was mixed with 20–40 mg doxorubicin hydrochloride (Hisun Pharmaceutical Co. LTD, Zhejiang, China) to create an emulsion that was subsequently injected into the tumor feeding arteries. Gelatin sponge particles (300–700 um, Cook, USA) were used to supplement embolization until the stagnation of artery flow appeared.

### Apatinib Administration

In the TACE-apatinib group, apatinib (500 mg/day) (Hengrui Pharmaceutical Co. Ltd, Jiangsu, China) was orally administrated 3–5 days after each TACE procedure. Apatinib dose adjustment was based on the tolerance of patients to the drug. The grading of adverse events associated with apatinib was conducted according to the National Cancer Institute Common Terminology Criteria for Adverse Events (version 4.0). If apatinib-related adverse events were equal to or greater than grade 3, then the dose of apatinib was reduced to 250 mg/day to alleviate or eliminate the adverse events. If these events (≥grade 3) didn't disappear after the dose adjustment, the administration of the drug was temporarily interrupted. The dose was resumed at 250 mg/day for patients who have experienced drug interruption when the adverse events had been alleviated or disappeared.

### Follow-Up

Follow-up of all patients was conducted through until January 2020. Follow-up contents included imaging examinations, such as abdominal contrast-enhanced CT or MRI, and laboratory tests, such as urine and blood routine test, liver function tests, and renal function analyses. The first follow-up was carried out at the end of the fourth week after the first TACE operation. A repeated TACE procedure was performed when the recurrent tumors or residual lesions were found by medical imaging. The next follow-up interval was extended to every 2 months starting at 4 weeks after the first TACE procedure.

### Assessments

Tumor response, overall survival (OS), and time to progression (TTP) were assessed. The medical records including CT, MRI, and follow-up data were reviewed. Treatment response was evaluated based on the Modified Response Evaluation Criteria in Solid Tumors (mRECIST) criteria (Lencioni and Llovet, 2010). All treatment response evaluations were assessed by a diagnostic radiologist (F.Y., with more than 15 years of experience) and an interventional radiologist (B.L., with more than 10 years of experience). Meanwhile, the treatment information and survival data of patients were blinded to them. Disease control rate (DCR) was defined as the portion of patients who achieved complete response (CR), partial response (PR), and stable disease (SD) (CR or PR or SD). OS was defined as the time from the first TACE to the last follow up or any reason death. TTP was defined as the time from the first TACE to the time that tumor progression.

### Statistical Analysis

All statistical analyses were performed by SPSS 24.0 software (IBM, Armonk, New York). Normally distributed data, non-normally distributed data and categorical variables were expressed as mean ± standard deviation, median (quartile range), and frequency (percentage), respectively. Kaplan-Meier method was used to describe the OS and TTP of the study cohort. Univariate analyses were implemented with a log-rank test (OS, TTP) and logistic regression (DCR). Variables with a value of *P* < 0.10 were entered into a multivariate analysis, which was performed by Cox proportional hazard regression model (OS, TTP) and logistic regression model (DCR). *P*-value < 0.05 (two-tailed) was statistically significant.

## Results

### Study Population

A total of 58 advanced HCC patients with APS were included in this study from June 2015 to January 2019. Twenty-seven patients underwent the treatment of TACE-apatinib, and 31 patients received TACE-alone treatment. The detailed baseline characteristics of the study population (gender, age, bilirubin, albumin, PT, ECOG, ascites, portal vein invasion, extrahepatic metastasis, AFP level, HBV infection, Child-Pugh class, TACE sessions) are listed in Table 1. The median follow-up period was 11 months (range, 3–30 months).

**Table 1:** Baseline characteristics of advanced HCC patients with APS.

**Characteristics**	**TACE-apatinib (*N* = 27) (No, %; Mean ± SD)**	**TACE alone (*N* = 31) (No, %; Mean ± SD)**	***P*-value**
**Gender**			0.107^a^
Male	24 (88.9%)	21 (67.7%)	
Female	3 (11.1%)	10 (32.3%)	
**Age (years)**	55.56 ± 5.2	58.65 ± 6.6	0.56^b^
**Bilirubin (μmol/L)**	17.2 ± 7.8	19.5 ± 13.8	0.45^b^
**Albumin (g/L)**	37.4 ± 4.9	38.1 ± 5.6	0.60^b^
**PT (s)**	13.9 ± 0.7	14.4 ± 2.0	0.20^b^
**ECOG**			0.79^a^
1	21 (77.8%)	25 (80.6%)	
2	6 (22.2%)	6 (19.4%)	
**Ascites**			0.99^a^
Yes	7 (25.9%)	8 (25.8%)	
No	20 (74.1%)	23 (74.2%)	
**Portal vein invasion**			0.83^a^
Yes	19 (70.4%)	21 (67.7%)	
No	8 (29.6%)	10 (32.3%)	
**Extrahepatic metastasis**			0.61^a^
**Yes**	14 (51.9%)	14 (45.2%)	
**No**	13 (48.1%)	17 (54.8%	
**Hepatitis**			0.76^a^
Hepatitis B	25 (92.6%)	28 (90.3%)	
Other	2 (7.4%)	3 (9.7%)	
**α-Fetoprotein level**			0.450^a^
>400 ng/mL	13 (48.1%)	18 (58.1%)	
≤ 400 ng/ml	14 (51.9%)	13 (41.9%)	
**Child-Pugh score**			0.75^a^
A	21 (77.8%)	23 (74.2%)	
B	6 (22.2%)	8 (25.8%)	
**TACE sessions**			0.85^a^
1	12 (44.4%)	13 (41.9%)	
2 or more	15 (55.6%)	18 (58.1%)	

*SD, Standard deviation; PT, Prothrombin time; ECOG, Eastern Cooperative Oncology Group; TACE, Transcatheter arterial chemoembolization*.

a *Chi-square test*.

b *Student's t-test*.

### The Efficacy of TACE-Apatinib for Advanced HCC With APS

In the TACE-apatinib group, there were no case with CR, 10(37.0%) cases with PR, 7 (25.9%) cases with SD, and 10 (37.0%) cases with progressive disease (PD). In the TACE-alone group, there were no case with CR, 5 (16.1%) cases with PR, 4 (12.9%) cases with SD, and 22 (71.0%) cases with PD. The DCR of tumor response was 62.9% in the TACE-apatinib group, which was significantly higher than 29.0% in the TACE-alone group (*P* = 0.01). Multivariate analysis showed that treatment method (TACE-apatinib) was an independent prognostic factor for DCR (Table 2).

**Table 2:** Univariate and multivariate analysis of prognostic factors for DCR.

**Variables**	**Univariate analysis**		**Multivariate analysis**	
	**HR (95% CI)**	***P*-value**	**HR (95% CI)**	***P*-value**
**Gender**
Male	1			
Female	0.465 (0.125, 1.730)	0.253		
**Age (years)**	0.956 (0.908, 1.007)	0.092	0.983 (0.926, 1.044)	0.581
**ECOG**
2	1			
1	0.769 (0.215, 2.747)	0.686		
**Ascites**
Yes	1			
No	1.304 (0.395, 4.306)	0.663		
**Portal vein invasion**
Yes	1			
No	0.703 (0.227, 2.183)	0.543		
**Extrahepatic**
**metastasis**
Yes	1			
No	1.167 (0.414, 3.290)	0.771		
**Bilirubin**	1.008 (0.963, 1.056)	0.721		
**Albumin**	0.955 (0.863, 1.056)	0.371		
**PT**	1.001 (0.710, 1.412)	0.995		
**Hepatitis**
Hepatitis B	1			
Other	0.280 (0.029, 2.674)	0.269		
**α-fetoprotein level**
>400 ng/mL	1			
≤ 400 ng/ml	0.440 (0.153, 1.266)	0.128		
**Child-Pugh score**
B	1			
A	0.519 (0.154, 1.754)	0.291		
**TACE sessions**
1	1			
2 or more	0.600 (0.210, 1.715)	0.340		
**Treatment method**
TACE-apatinib	1			
TACE alone	4.156 (1.382, 12.493)	**0.011**	3.517 (1.019, 12.147)	**0.047**

*DCR, Disease control rate; HR, Hazard ratio; CI, Confidence interval; ECOG, Eastern Cooperative Oncology Group; PT, Prothrombin time; TACE, Transcatheter arterial chemoembolization*.

*Statistical analysis: Logistic regression*.

*The bold values means that the value of P < 0.05*.

**Figure 1:**
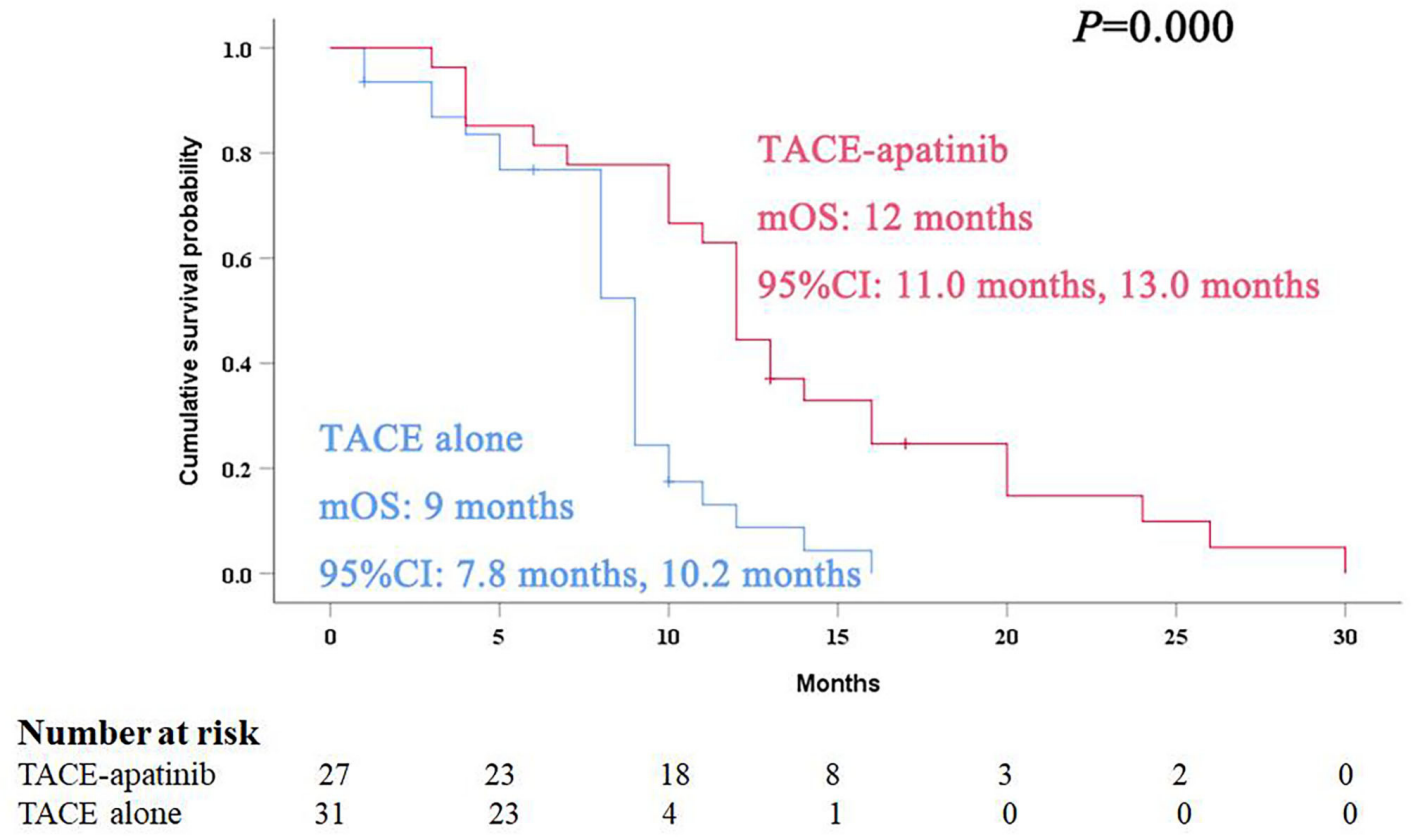
Kaplan–Meier curves of overall survival for advanced hepatocellular carcinoma patients with hepatic arterioportal shunts who received the treatment of transarterial chemoembolization (TACE)-apatinib.

**Figure 2:**
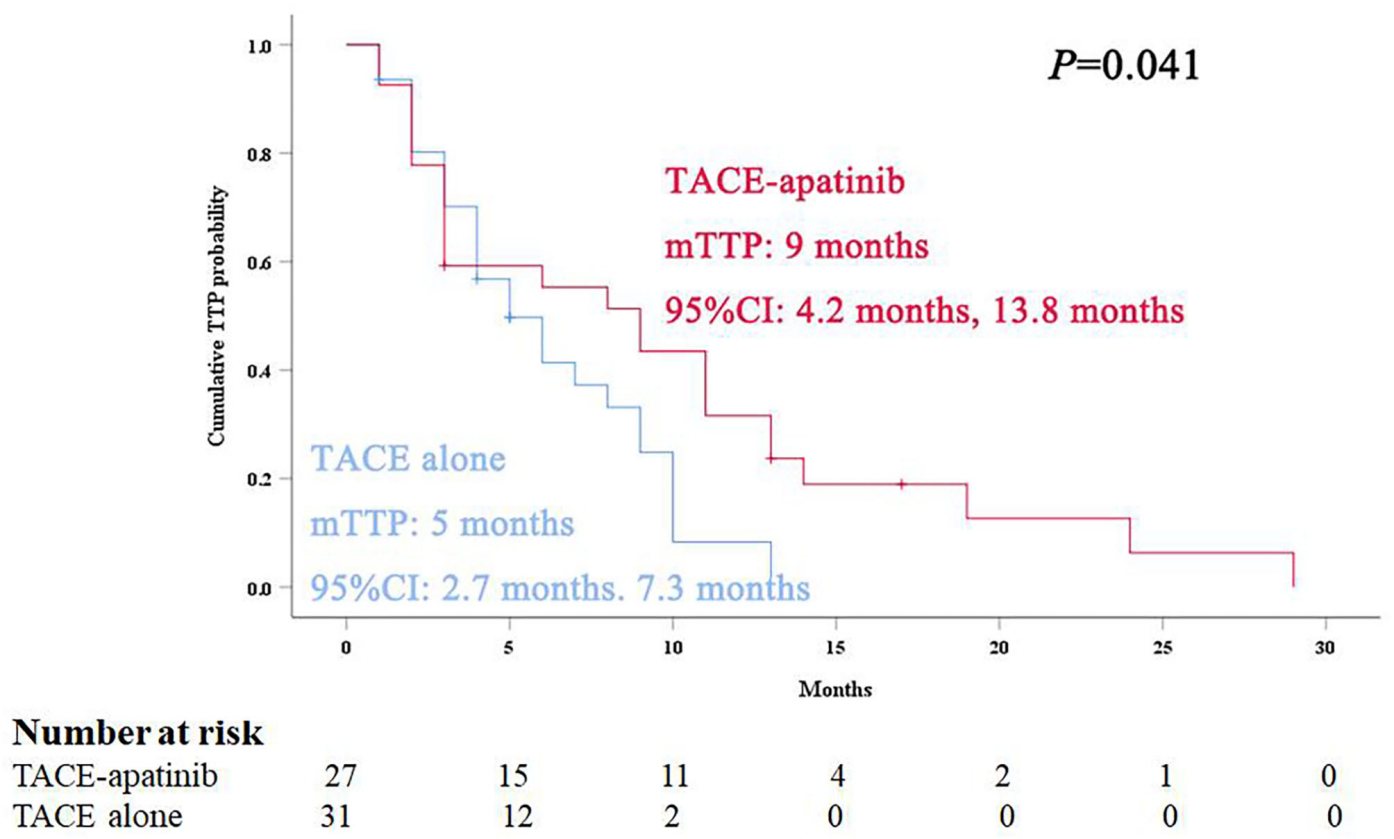
Kaplan–Meier curves of time to progression for advanced hepatocellular carcinoma patients with hepatic arterioportal shunts who received the treatment of transarterial chemoembolization (TACE)-apatinib.

During the follow-up, 24 patients (88.9%) died in the TACE-apatinib group, and 27 patients (87.1%) died in the TACE-alone group. The main causes of death were liver failure and gastrointestinal bleeding. The median overall survival (OS) in the TACE-apatinib group was 12.0 months (95% CI: 11.0, 13.0 months), and in the TACE alone group was 9.0 months (95% CI: 7.8, 10.2 months) (Figure 1). The median OS between the two groups was significantly different. The median TTP in the TACE-apatinib group was 9.0 months (95% CI: 4.2, 13.8 months), which was significantly longer than 5.0 months (95% CI: 2.7, 7.3 months) in the TACE-alone group (*P* = 0.041) (Figure 2).

### Prognostic Factors Affecting OS and TTP

Univariate analyses showed that portal vein invasion, TACE sessions, and treatment method were correlated with OS (Table 3). Then, through multivariable analyses, we found that TACE-apatinib was a protective factor for OS (Table 4). Univariate analyses showed that albumin and PT were significantly associated with TTP (Table 3), but there was no independent risk factor in multivariate analyses for TTP (Table 5).

**Table 3:** Univariate analysis of prognostic factors for overall survival and time to progression.

**Variables**	**OS**		**TTP**	
	**HR (95% CI)**	***P*-value**	**HR (95% CI)**	***P*-value**
**Gender**
Male	1		1	
Female	0.681 (0.199, 2.328)	0.540	0.636 (0.183, 2.205)	0.475
**Age (years)**	0.979 (0.937, 1.023)	0.350	0.993 (0.952, 1.036)	0.741
**ECOG**
2	1		1	
1	0.904 (0.332, 2.457)	0.843	0.717 (0.264, 1.952)	0.516
**Ascites**
Yes	1		1	
No	0.861 (0.334, 2.215)	0.756	1.183 (0.457, 3.065)	0.729
**Portal vein**
**invasion**
Yes	1		1	
No	2.794 (1.065, 7.331)	**0.037**	2.027 (0.780, 5.270)	0.147
**Extrahepatic**
**metastasis**
Yes	1		1	
No	0.938 (0.411, 2.142)	0.880	1.118 (0.482, 2.597)	0.794
**Bilirubin**	1.032 (0.972, 1.096)	0.300	1.014 (0.956, 1.076)	0.641
**Albumin**	1.022 (0.944, 1.107)	0.592	1.086 (0.996, 1.185)	**0.032**
**PT**	0.763 (0.441, 1.320)	0.333	0.508 (0.255, 1.012)	**0.044**
**Hepatitis**
Hepatitis B	1		1	
Other	1.427 (0.329, 6.200)	0.635	0.459 (0.100, 2.100)	0.316
**α-fetoprotein level**
>400 ng/mL	1		1	
≤ 400 ng/ml	1.199 (0.535, 2.688)	0.659	1.301 (0.568, 2.977)	0.534
**Child-Pugh score**
B	1		1	
A	0.996 (0.361, 2.749)	0.994	1.086 (0.397, 2.971)	0.873
**TACE sessions**
1	1		1	
2 or more	2.031 (0.880, 4.688)	**0.047**	1.820 (0.754, 4.394)	0.183
**Treatment method**
TACE-apatinib	1			
TACE alone	2.918 (1.573, 5.415)	**0.001**	1.787 (0.978, 3.266)	0.059

*OS, Overall survival; TTP, Time to progression; HR, Hazard ratio; CI, Confidence interval; ECOG, Eastern Cooperative Oncology Group; PT, Prothrombin time; TACE, Transcatheter arterial chemoembolization*.

*Statistical analysis: log-rank test*.

*The bold values means that the value of P < 0.05*.

**Table 4:** Multivariate analysis of prognostic factors for overall survival.

**Variables**	**HR (95% CI)**	***P*-value**
**Portal vein invasion**
Yes	1	
No	0.835 (0.437, 1.596)	0.585
**TACE sessions**
1	1	
2 or more	0.716 (0.405, 1.266)	0.251
**Treatment method**
TACE-apatinib	1	
TACE alone	2.683 (1.441, 4.994)	**0.002**

*HR, Hazard ratio; CI, Confidence interval; TACE, Transcatheter arterial chemoembolization*.

*Statistical analysis: Cox proportional hazards regression model*.

**Table 5:** Multivariate analysis of prognostic factors for time to progression.

**Variables**	**HR (95% CI)**	***P*-value**
**Albumin (g/L)**	1.036 (0.969, 1.106)	0.301
**PT (s)**	0.946 (0.770, 1.163)	0.599
**Treatment method**
TACE-apatinib	1	
TACE alone	1.736 (0.909, 3.314)	0.095

*HR, Hazard ratio; CI, Confidence interval; PT, Prothrombin time; TACE, Transcatheter arterial chemoembolization*.

*Statistical analysis: Cox proportional hazards regression model*.

*The bold values means that the value of P < 0.05*.

### Adverse Events Related to TACE or Apatinib

**Figure 3:**
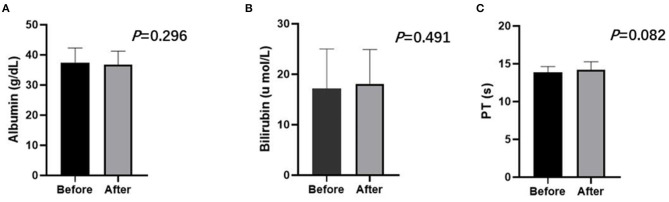
Liver function including albumin **(A)**, bilirubin **(B)**, PT **(C)** of the TACE-apatinib group before and after treatment at 4 weeks. Paired sample *T*-test showed no statistic difference (*P* > 0.05).

No treatment-related deaths were observed in this study. Three representative indicators of liver function (bilirubin, albumin, and PT) were observed at 4 weeks after the first TACE-apatinib treatment, and did not differ significantly from the baseline values at this time point (Figures 3A–C). The detailed adverse events that related to TACE were listed in Table 6. There were 2 (7.4%) patients who had adverse events of hepatorenal syndrome or hepatic arterial dissection after TACE in the TACE-apatinib group. In the TACE-alone group, only 1 patient experienced adverse event of hepatic arterial dissection. There was no significant difference in the adverse events that related to TACE between the two groups. The adverse events that related to apatinib were listed in the Table 7. The apatinib-related adverse events in the TACE-apatinib group occurred in 24 (88.9%) out of the 27 patients. Four (14.8%) patients developed grade 3 adverse events, and its duration was 1–2 weeks. The symptoms that related to these adverse events in the patients were alleviated or eliminated after drug reduction or interruption and symptomatic treatments. There was no occurrence of grade 4 and grade 5 adverse events. These symptoms were alleviated within 7 days after treatment and no further complications occurred.

**Table 6:** Adverse events related to TACE.

**Adverse events**	**TACE-apatinib (*n* = 27)**	**TACE alone (*n* = 31)**	***P*-value**
Hepatorenal syndrome	1 (3.7%)	0 (0%)	0.944
Inguinal hematoma	0 (0%)	0 (0%)	–
Hepatic arterial dissection	1 (3.7%)	1 (3.2%)	1
Pulmonary oil embolization	0 (0%)	0 (0%)	–

*TACE, Transcatheter arterial chemoembolization*.

*Statistical analysis: Chi-square test*.

**Table 7:** Adverse events related to apatinib in the TACE–apatinib group.

**Adverse events**	**Grade 1**	**Grade 2**	**Grade 3**	**Grade 4**	**Grade 5**	**All events**
Hand foot skin reactions	9 (33.3%)	8 (29.6%)	3 (11.1%)	0 (0%)	0 (0%)	20 (74.1%)
Hypertension	6 (22.2%)	7 (25.9%)	1 (3.7%)	0 (0%)	0 (0%)	14 (51.8%)
Diarrhea	6 (22.2%)	4 (14.8%)	0 (0%)	0 (0%)	0 (0%)	10 (37.0%)
Fatigue	5 (18.5%)	3 (11.1%)	0 (0%)	0 (0%)	0 (0%)	8 (29.6%)
Oral ulcer	2 (7.4%)	1 (3.7%)	0 (0%)	0 (0%)	0 (0%)	3 (11.1%)
Voice change	2 (7.4%)	0 (0%)	0 (0%)	0 (0%)	0 (0%)	2 (7.4%)
Proteinuria	2 (7.4%)	7 (25.9%)	0 (0%)	0 (0%)	0 (0%)	9 (33.3%)
Gastrointestinal hemorrhage	3 (11.1%)	1 (3.7%)	0 (0%)	0 (0%)	0 (0%)	4 (14.8%)

*TACE, Transcatheter arterial chemoembolization*.

## Discussion

TACE is the most widely used and an effective conservative treatment for unresectable HCC (European Association for the Study of the Liver, 2018). Emerging studies have revealed that the combination treatment of TACE with sorafenib can benefit advanced HCC patients (Bai et al., 2013; Choi et al., 2013; Zhao et al., 2013), and apatinib functions via similar mechanisms to facilitate HCC treatment (Fontanella et al., 2014). APS is frequently observed in patients with HCC (Wu et al., 2018). The presence of APS affects the survival of HCC patients and the safety of TACE procedure (Murata et al., 2005, 2009). Therefore, timely and complete embolization of shunts before the TACE procedure may represent a suitable treatment approach for advanced HCC with APS.

Few studies to date have reported on use of TACE-apatinib for the treatment of advanced HCC patients with APS. We found that for advanced HCC patients with APS who underwent TACE-apatinib, the median OS was 12.0 months, the median TTP was 9.0 months, and the DCR was 62.9%, with these values being significantly greater than those of the TACE-alone group. Some previous studies have focused on the combined treatment of TACE with apatinib as a means of improving treatment efficacy of advanced HCC (Qiu et al., 2019; Zhu et al., 2019). Kan et al. (2020) reported 90 advanced HCC patients were treated with TACE-apatinib, and the results showed that the median OS was 14.0 months and the median TTP was 7.0 months. Although the results of our study were slightly inferior to Kan et al.'s study, it is acceptable considering that all the patients in our study were with APS. Based on the results of our study, it is believed that TACE-apatinib treatment may benefit advanced HCC patients with APS.

APS may affect the safety of TACE as lipiodol can easily pass through the shunts, potentially resulting in the embolization of normal liver tissue or pulmonary embolism (Ziessman et al., 1984). However, transcatheter arterial occlusion of APS is technically feasible (Chen et al., 2014). We found that performing the TACE procedure after complete shunt embolization was well-tolerated for advanced HCC with APS in both groups, with no patients having experienced pulmonary embolization as a consequence of treatment. Common complications of TACE in this study were fever, abdominal pain, and elevated total bilirubin, consistent with prior reports (Choi et al., 2013; Kan et al., 2020). There were no significant differences in patient liver function when comparing pre- and post-treatment levels at 4 weeks after initial TACE treatment in the TACE-apatinib group, indicating that injury to normal liver tissue was limited. In line with other studies, our study revealed that apatinib was well-tolerated by advanced HCC patients with APS (Yang et al., 2019; Kan et al., 2020). The observed apatinib-related adverse events in our study were predominantly grade 1 or 2, and the symptoms that were related to these adverse events in the patients were alleviated or eliminated after drug reduction or interruption and symptomatic treatments.

There were some limitations to our study. First, this was a retrospective study with a small sample size. Second, our study data came from a single center. It is therefore necessary that prospective multicenter clinical trials be conducted in the future to validate our findings.

In conclusion, our study revealed that TACE-apatinib was safe and effective for advanced HCC patients with APS, and apatinib improved the efficacy of TACE in the treatment of these patients.

## Data Availability Statement

The raw data supporting the conclusions of this article will be made available by the authors, without undue reservation.

## Ethics Statement

The studies involving human participants were reviewed and approved by Wuhan Union Hospital. The patients/participants provided their written informed consent to participate in this study. Written informed consent was obtained from the individual(s) for the publication of any potentially identifiable images or data included in this article.

## Author Contributions

TS, YR, XK, FY, and CZ: conceptualization. YR and LC: methodology. TS, YR, and WZ: validation. TS and CZ: formal analysis. TS: writing—original draft preparation. YR, XK, FY, and CZ: writing—review and editing. TS, YR, and XK: visualization. FY and CZ: supervision. All authors have read and agreed to the published version of the manuscript.

## Conflict of Interest

The authors declare that the research was conducted in the absence of any commercial or financial relationships that could be construed as a potential conflict of interest.

## Abbreviations

HCC: hepatocellular carcinoma
BCLC: Barcelona Clinic Liver Cancer
OS: overall survival
TACE: transarterial chemoembolization
TACE-apatinib: TACE combining with apatinib
ECOG: Eastern Cooperative Group Performance Status
CT: computed tomography
MR: magnetic resonance
AFP: a-Fetoprotein
mRECIST: modified Response Evaluation Criteria in solid tumors
PR: Partial response
PD: Progressive disease
SD: Stable disease
DCR: disease control rate
TTP: time to tumor progression
APS: hepatic Arterioportal shunts
CI: Confidence interval.

## References

[B1] Bai W. Wang Y. J. Zhao Y. Qi X. S. Yin Z. X. He C. Y.. (2013). Sorafenib in combination with transarterial chemoembolization improves the survival of patients with unresectable hepatocellular carcinoma: a propensity score matching study. J. Digit. Dis. 14, 181–190. 10.1111/1751-2980.12038 23324079

[B2] Bray F. Ferlay J. Soerjomataram I. Siegel R. L. Torre L. A. Jemal A. (2018). Global cancer statistics 2018: GLOBOCAN estimates of incidence and mortality worldwide for 36 cancers in 185 countries. Cancer J. Clin. 68, 394–424. 10.3322/caac.21492 30207593

[B3] Chen J. Chen S. Xi W. Wu B. Yu H. Gao Y. (2014). Transcatheter arterial chemoembolization and chemotherapy plus sorafenib in a large hepatocellular carcinoma with arterioportal shunt. Case Rep. Oncol. Med. 2014:392403. 10.1155/2014/392403 25431715 PMC4241331

[B4] Choi G. H. Shim J. H. Kim M. J. Ryu M. H. Ryoo B. Y. Kang Y. K.. (2013). Sorafenib alone vs. sorafenib combined with transarterial chemoembolization for advanced-stage hepatocellular carcinoma: results of propensity score analyses. Radiology 269, 603–611. 10.1148/radiol.13130150 23864102

[B5] Ding J. Chen X. Gao Z. Dai X. Li L. Xie C.. (2013). Metabolism and pharmacokinetics of novel selective vascular endothelial growth factor receptor-2 inhibitor apatinib in humans. Drug Metab. Dispo. 41, 1195–1210. 10.1124/dmd.112.050310 23509226

[B6] European Association for the Study of the Liver. (2018). EASL clinical practice guidelines: management of hepatocellular carcinoma. J. Hepatol. 70:817. 10.1016/j.jhep.2019.01.020 29628281

[B7] Fontanella C. Ongaro E. Bolzonello S. Guardascione M. Fasola G. Aprile G.. (2014). Clinical advances in the development of novel VEGFR2 inhibitors. Ann. Transl. Med. 2:123. 10.3978/j.issn.2305-5839.2014.08.14 25568876 PMC4260048

[B8] Forner A. Reig M. E. de Lope C. R. Bruix J. (2010). Current strategy for staging and treatment: the BCLC update and future prospects. Semin. Liver Dis. 30, 61–74. 10.1055/s-0030-1247133 20175034

[B9] Kan X. Liang B. Zhou G. Xiong B. Pan F. Ren Y.. (2020). Transarterial chemoembolization combined with apatinib for advanced hepatocellular carcinoma: a propensity score matching analysis. Front. Oncol. 10:970. 10.3389/fonc.2020.00970 32733791 PMC7358575

[B10] Kim Y. J. Lee H. G. Park J. M. Lim Y. S. Chung M. H. Sung M. S.. (2007). Polyvinyl alcohol embolization adjuvant to oily chemoembolization in advanced hepatocellular carcinoma with arterioportal shunts. Korean J. Radiol. 8, 311–319. 10.3348/kjr.2007.8.4.311 17673842 PMC2627160

[B11] Lencioni R. Llovet J. M. (2010). Modified RECIST (mRECIST) assessment for hepatocellular carcinoma. Semin. Liver Dis. 30, 52–60. 10.1055/s-0030-1247132 20175033 PMC12268942

[B12] Lencioni R. Llovet J. M. Han G. Tak W. Y. Yang J. Guglielmi A. (2016). Sorafenib or placebo plus TACE with doxorubicin-eluting beads. for intermediate stage HCC: the SPACE trial. J. Hepatol. 64, 1090–1098. 10.1016/j.jhep.2016.01.012 26809111

[B13] Lu W. Jin X. L. Yang C. Du P. Jiang F. Q. Ma J. P.. (2017). Comparison of efficacy between TACE combined with apatinib and TACE alone in the treatment of intermediate and advanced hepatocellular carcinoma: a single-center randomized controlled trial. Cancer Biol. Ther. 18, 433–8. 10.1080/15384047.2017.1323589 28548587 PMC5536939

[B14] Murata S. Tajima H. Abe Y. Fukunaga T. Nakazawa K. Mohamad R. A.. (2005). Temporary occlusion of two hepatic veins for chemoembolization of hepatocellular carcinoma with arteriohepatic vein shunts. AJR 184, 415–7. 10.2214/ajr.184.2.01840415 15671355

[B15] Murata S. Tajima H. Nakazawa K. Onozawa S. Kumita S. Nomura K. (2009). Initial experience of transcatheter arterial chemoembolization during portal vein occlusion for unresectable hepatocellular carcinoma with marked arterioportal shunts. Eur. Radiol. 19, 2016–23. 10.1007/s00330-009-1349-y 19238387

[B16] Okuda K. Musha H. Yamasaki T. Jinnouchi S. Nagasaki Y. Kubo Y.. (1977). Angiographic demonstration of intrahepatic arterioportal anastomoses in hepatocellular carcinoma. Radiology 122, 53–58. 10.1148/122.1.53 186844

[B17] Qiu Z. Shen L. Chen S. Qi H. Cao F. Xie L.. (2019). Efficacy of apatinib in transcatheter arterial chemoembolization (TACE) refractory intermediate and advanced-stage hepatocellular carcinoma: a propensity score matching analysis. Cancer Manag. Res. 11, 9321–9330. 10.2147/CMAR.S223271 31802950 PMC6830366

[B18] Velazquez R. F. Rodriguez M. Navascues C. A. Linares A. Perez R. Sotorrios N. G.. (2003). Prospective analysis of risk factors for hepatocellular carcinoma in patients with liver cirrhosis. Hepatology 37, 520–7. 10.1053/jhep.2003.50093 12601348

[B19] Wang Y. Tang Z. (2018). A novel long-sustaining system of apatinib for long-term inhibition of the proliferation of hepatocellular carcinoma cells. Onco Targets Ther. 11, 8529–41. 10.2147/OTT.S188209 30555243 PMC6278711

[B20] Wu H. Zhao W. Zhang J. Han J. Liu S. (2018). Clinical characteristics of hepatic arterioportal shunts associated with hepatocellular carcinoma. BMC Gastroenterol. 18:174. 10.1186/s12876-018-0899-3 30419830 PMC6233279

[B21] Xue J. M. Astere M. Zhong M. X. Lin H. Shen J. Zhu Y. X. (2018). Efficacy and safety of apatinib treatment for gastric cancer, hepatocellular carcinoma and non-small cell lung cancer: a meta-analysis. Onco Targets Ther. 11, 6119–28. 10.2147/OTT.S172717 30288047 PMC6160267

[B22] Yang C. Qin S. (2018). Apatinib targets both tumor and endothelial cells in hepatocellular carcinoma. Cancer Med. 7, 4570–83. 10.1002/cam4.1664 30109780 PMC6144148

[B23] Yang Z. Chen G. Cui Y. Xiao G. Su T. Yu J.. (2019). The safety and efficacy of TACE combined with apatinib on patients with advanced hepatocellular carcinoma: a retrospective study. Cancer Biol. Ther. 20, 321–7. 10.1080/15384047.2018.1529099 30332553 PMC6370378

[B24] Zhao S. Zhang T. Dou W. Wang E. Wang M. Wang C.. (2020). A comparison of transcatheter arterial chemoembolization used with and without apatinib for intermediate- to advanced-stage hepatocellular carcinoma: a systematic review and meta-analysis. Ann. Transl. Med. 8:542. 10.21037/atm.2020.02.125 32411765 PMC7214911

[B25] Zhao Y. Wang W. J. Guan S. Li H. L. Xu R. C. Wu J. B.. (2013). Sorafenib combined with transarterial chemoembolization for the treatment of advanced hepatocellular carcinoma: a large-scale multicenter study of 222 patients. Ann. Oncol. 24, 1786–92. 10.1093/annonc/mdt072 23508822

[B26] Zhu Y. Feng B. Mei L. Sun R. Guo C. Zhu J. (2019). Clinical efficacy of TACE combined with apatinib in the treatment of advanced hepatocellular carcinoma. J. BUON 24, 608–614. 31128013

[B27] Ziessman H. A. Thrall J. H. Yang P. J. Walker S. C. Cozzi E. A. Niederhuber J. E.. (1984). Hepatic arterial perfusion scintigraphy with Tc-^99^m-MAA: use of a totally implanted drug delivery system. Radiology 152, 167–172. 10.1148/radiology.152.1.6233632 6233632

